# Participation and argument in legislative debate on statewide smoking restrictions

**DOI:** 10.1186/1478-4505-5-12

**Published:** 2007-10-22

**Authors:** Dorie E Apollonio, Peggy Lopipero, Lisa A Bero

**Affiliations:** 1 Department of Clinical Pharmacy, University of California, San Francisco, San Francisco, California, US; 2 Biology Department, City College of San Francisco, San Francisco, California, US

## Abstract

**Background:**

In this paper we review the relationship between participation in legislative hearings, the use of ideological arguments, and the strength of public health legislation using a theoretical construct proposed by E. E. Schattschneider in 1960. Schattschneider argued that the breadth and types of participation in a political discussion could change political outcomes.

**Methods:**

We test Schattschneider's argument empirically by reviewing the efforts of six states to pass Clean Indoor Air Acts by coding testimony given before legislators, comparing these findings to the different characteristics of each state's political process and the ultimate strength of each state's legislation.

**Results:**

We find that although greater participation is associated with stronger legislation, there is no clear relationship between the use and type of ideological arguments and eventual outcomes.

**Conclusion:**

These findings offer validation of a long-standing theory about the importance of political participation, and suggest strategies for public health advocates seeking to establish new legislation.

## Background

This paper considers the question of whether participation is associated with the passage of public health legislation. To do so, we review workplace smoking legislation proposed and debated in six different US states, including the extent of outside participation, which we define for the purposes of this study as the decision by individuals to give spoken or written testimony before the legislature in support of or opposition to a proposed policy change.

Nearly fifty years ago political scientist E. E. Schattschneider (1960)[1] theorized that political outcomes could be explained by considering the number of participants and the types of arguments used by advocates for change and those used by individuals and groups that preferred the status quo. Given that full societal participation in politics is rare, Schattschneider believed that groups that benefited from the status quo, primarily businesses and wealthy individuals, controlled the political environment. His claims have been cited repeatedly in studies of political and legislative behavior, guiding much of the research on political influence [2-4] and social movements[5,6]

However, the empirical question of whether these assumptions were correct remains unanswered; this was never empirically validated in Schattschneider's work, and it has not drawn much scholarly attention, possibly because, as Tversky and Kahneman (1974) noted, researchers also tend to accept theoretically intuitive results as proven after only limited study[7] Research in political science and public health has addressed related questions; showing, for example, that increased media attention to an issue leads to more Congressional hearings and increased political participation at the national level,[8] and also appears to increase policy making activity (although changes may not be effective)[9] Case studies of US federal and state government efforts to pass tobacco control legislation suggest that they succeed or fail in large part based on the degree to which public health advocates were engaged in the political process [10-18] Case studies of similar efforts in other countries also reveal the importance of participation by health advocates, but as with all case studies, these results are not comparative [19-26] Yet despite the existence of such case studies, which in the US extend to a range of issues, [27-29] much of the theoretical research in political science on the use of testimony and expertise in policy making claims that politicians largely ignore testimony, and instead support policies that are easy to explain to constituents and as a result are unlikely to address complicated problems effectively [30-34] However, recent research suggests such pessimism is unwarranted,[35,36] and our findings extend on this work by suggesting specific tactics that may lead to more socially desirable outcomes.

The question of whether the number of participants and types of argument predict the outcome of a political debate is particularly relevant to public health legislation, which is collective in nature because it seeks to establish benefits that are available to anyone without restriction. It is exemplified in efforts to decrease the use of tobacco, which, as a product responsible for 440,000 deaths a year, is the leading cause of mortality in the United States[37] Although tobacco control advocates in the US have frequently been successful on the local level, the tobacco industry has strong ties to many federal and state legislators and often forestalls or weakens proposed new legislation[11,12,38,39] If Schattschneider was correct, prospective public health legislation is most likely to benefit from outside participation, that is, participation by non-legislators. Establishing a relationship between outcomes and participation may be useful for public health advocates in determining where to focus political effort. Schattschneider argued that greater participation was responsible for changing the status quo. However, it is also possible that increased outside participation may not cause stronger legislation protecting public health, but is a necessary condition to its passage. In either case, public health advocates may find that it is not worth pursuing legislation without broad public support.

This kind of strategic thinking is particularly relevant in cases where there is known opposition that benefits from the status quo. Public health advocacy frequently confronts existing corporate practices,[40] and corporate behavior is increasingly recognized as a fundamental cause of disease[41,42] Corporate activity is implicated in health problems such as obesity, tobacco, alcohol, and other drug use, as well as in risks posed by automobiles, guns, and pharmaceuticals[42] Efforts to change health-damaging corporate behavior are key emerging issues in public health advocacy. Tobacco control advocacy has been the leading edge of public health efforts to change corporate practices that affect public health,[42] and its successes and failures offer insight for future public health campaigns. Increasingly, public health researchers are seen as having an obligation to be involved in policy and media efforts to improve public health[43] and to deal with the controversy inherent in questioning the status quo[44]

In attempting to pass restrictions on smoking, public health advocates face a highly organized and well-funded industry that strongly resists regulation[11] However, in 1992, the US Environmental Protection Agency (EPA) released a report on the health effects of secondhand smoke,[45] which concluded that secondhand smoke was carcinogenic. This triggering event led many US states to contemporaneously consider regulating workplace smoking. Different states proposed a variety of policies, and each had different protocols for considering legislation, ranging from legislative hearings to a task force soliciting public comment. As a result, state legislation on this issue offers a wide range of outcomes and participation levels for comparison.

Our analysis of state legislative efforts to pass workplace smoking restrictions builds on previous research considering state regulatory efforts to restrict smoking. Participation in regulatory hearings on workplace smoking has been dominated by the tobacco industry, with little or no participation by public health advocates [46-50] Historically, regulatory hearings, unlike legislative hearings, were closed to the public[51,52] Relevant industry representatives were invited to present testimony, however, and were traditionally considered to be scientifically objective in presenting evidence about potential regulation[53] Regulators no longer believe that industry scientists are objective and recognize the vulnerability of regulatory hearings to "Astroturf" lobbying – the phenomenon where an interest group floods a hearing with form letters in support of its preferred position[54] However, legislative hearings have different characteristics than regulatory hearings, and these differences make legislative hearings far more likely to draw broad-based participation[55] Legislative hearings typically solicit spoken testimony rather than, or in addition to, written testimony. State  legislatures also frequently hold open rather than closed hearings. The tradition of holding hearings that can be attended by non-legislators, in combination with the expectation that individuals will appear in person to give testimony, may make legislative hearings less susceptible to industry efforts to hijack public commentary.

### Theory

We base our analysis on arguments proposed by Schattschneider in *The Semisovereign People *(1960), taking three hypotheses and applying them to the case of workplace smoking legislation. First, Schattschneider suggested that the scope of argument would expand if the group that is losing an argument, or proposing to change the status quo, can call in reinforcements, which he referred to as "the crowd"[1] He wrote, "the outcome of every conflict is determined by the *extent *to which the audience becomes involved in it"[1] (emphasis in original). Because there is a difference between the number of people who may be *affected *by a political action and the number of people who *participate*, an outcome cannot be predicted in advance by measuring the strength of one interest relative to another. However, more participation should advance the public interest, because the public is always the largest group affected. By this argument, states that passed stronger workplace smoking legislation should also have had more outside participation in legislative debate.

Hypothesis 1. Stronger workplace smoking legislation will be associated with more outside participation in legislative debate.

Schattschneider also proposed that the patterns of debate would predict policy outcomes. He believed that participants seeking to restrict the scope of conflict would emphasize ideological arguments and procedural questions (for example, whether or not the government has the right to regulate) rather than claims of fact, such as scientific evidence[1] With workplace smoking restrictions, public health advocates started from a position of relative disadvantage given that there was little existing regulation and the tobacco industry had traditionally cultivated state government. Arguments that focused on non-scientific ideological claims like whether or not government had the right to regulate would advantage the tobacco industry, because they are not objectively right or wrong. These kinds of claims should be more appealing to industry in part because they cannot be disproved. The tobacco industry did in fact focus on these kinds of arguments in its efforts to prevent workplace smoking *regulation*[46] Our second hypothesis is that the use of ideological arguments, relative to other kinds of arguments, is related to the strength of legislation.

Hypothesis 2. Stronger workplace smoking legislation will be associated with less focus on ideological arguments.

In considering the scope of political debate, Schattschneider also argued that ideological arguments come in two forms: One type "privatizes" conflict and the other "socializes" conflict[1] He noted that privatizing arguments were typically ideological in nature, and claimed that the issue in question should be outside the scope of governmental action. Arguments that emphasize individualism or free enterprise suggest that outside forces, such as economic self-interest, will serve in lieu of government to accomplish the same policy goals,[1] or alternatively, that the underlying values they represent are superior to the goals of collective regulation. These types of arguments are often associated with beliefs about the appropriate role of government, and lead actors to behave in ways that might seem counter to their own personal interests. Individuals who have an ideological commitment to the limited role of government may, for example, oppose restrictions on smoking even though they themselves are non-smokers and do not like to be around cigarette smoke[56]

In contrast, Schattschneider noted that socializing arguments suggest that the nature of the debate requires government to get involved, either because not doing so will impose costs on society, or because the underlying values they represent justify social regulation. These arguments can include claims of threats to the public (in the case of tobacco, public health), civil rights, justice, or claims of equal protection[1] Socializing arguments are often associated with beliefs about the appropriate role of government. They assume that government should take action to improve the lives of individuals. The predicted effects of privatizing and socializing arguments, which largely measure support and opposition to legislation across ideological arguments, are summarized in our third and final hypothesis.

Hypothesis 3. In the context of ideological arguments, stronger workplace smoking legislation will be associated with a greater share of socializing arguments, and a lesser share of privatizing arguments.

## Methods

This paper specifically considers the efforts of six different American states to pass Clean Indoor Air Acts between 1992 and 2003. In 1992, the US EPA released a risk assessment on the health effects of secondhand smoke called *The Respiratory Health Effects of Passive Smoking: Lung Cancer and Other Disorders*[45] In this report, the EPA labeled secondhand smoke a Class A carcinogen for the first time. This evidence catalyzed several states to introduce and debate legislation that would restrict workplace smoking, a major source of secondhand smoke exposure.

We searched the American Lung Association's database "State Legislated Action on Tobacco Issues" and Lexis/Nexis to identify states that adopted or amended legislation to restrict tobacco use in private workplaces, including restaurants and bars. We also looked for states where proposed legislation failed. After contacting the legislative research offices of each state that passed legislation between 1992 and 2002, we found records of legislative proceedings were available for Florida, Louisiana, Oregon, South Dakota, and Utah. To identify those states where legislative attempts failed, we contacted the tobacco control officers and each regional American Lung Association office in the twenty-five states that by 2002 had no policy in place. (Contact information was graciously provided by Chris Bostic, Manager of Policy Analysis at the American Lung Association and Kristen Tertzakian, Senior Analyst of Tobacco Control Policy at the Association of State and Territorial Health Officials.) Six states had failed bills that would have established a statewide policy stronger than existing local restrictions, but only North Dakota had maintained records of its legislative proceedings. Although this set of states is incomplete, it includes a range of regions and types of legislatures that suggest our results can be generalized to the rest of the United States.

We collected all available legislative records for each of the six states. The data included audiotapes of committee hearings and floor debates that we transcribed verbatim, all versions of each bill introduced (original, engrossed, and enrolled), the text of any amendments offered during the proceedings, any written testimony regarding the proposed pieces of legislation, attendance and voting records, and any legislative meeting minutes. For Florida, we elected to include data from legislative proceedings dating back to 1985, because legislative testimony from the 1990s frequently referenced issues raised in the earlier debates. A year prior to the enactment of its first indoor air restrictions, the Utah legislature appointed a taskforce to study issues regarding environmental tobacco smoke and to recommend state action regarding these issues. We collected the audiotapes from each of the taskforce meetings as well as letters submitted to the taskforce and included them as data for the Utah case study.

We conducted a content analysis of all available oral and written testimony for both legislators and non-legislative participants. If both oral and written testimony were submitted by the same participant, the data providing the most extensive arguments were selected for analysis. Each document was coded for the participant's position on the legislation, the affiliation of the participant, and the arguments made. We developed coding categories for the arguments inductively and in conjunction with previously constructed coding instruments used in studies of regulatory proceedings [46-48] We defined the smallest text unit as a sentence and each text unit could be coded for multiple occurrences of an argument. Categories of argument included science/health effects, economic, ideological, and governmental/implementation, and some claims were coded under more than one category. Multiple researchers coded the documents with each coder responsible for a different set of argument types. When all coding had been completed, the full research team reviewed the work as a quality control measure.

To rank the relative strength of each state's smoking restrictions we applied a modified version of a rating system of state clean indoor air laws developed by the National Cancer Institute's State Cancer Legislative Database Program[57] The rating system identifies state specific smoking restrictions in seven indoor settings and scores them according to the extent to which such restrictions minimize exposure to secondhand smoke. It also scores elements of the state laws that narrow or limit its application, whether the law defines penalties and enforcement, or if the law preempts those of lower jurisdictions. We calculated scores for the extent of restrictions in private workplaces, restaurants, and bars and scored for any limitations or exemptions, penalties/enforcement, and preemption, with a higher score representing more public health protection, which we refer to as stronger legislation.

Our expectations relate to *outside *participation in the legislative process, so our results only review the testimony of *non-legislators*. Our analysis relies primarily on the set of codes designated as ideological arguments. We identified the direction of ideological arguments as being either privatizing or socializing. Ideological arguments included the kinds of statements that Schattschneider identified as privatizing, such as "individualism, free private enterprise, privacy"[1] and variants of these arguments that apply only to the issue of smoking, such as smokers' rights and courtesy to smokers. They also included claims Schattschneider identified as socializing, such as "equal protection of the laws, justice"[1] and variants of these arguments that apply only to the issue of smoking, such as claims about nonsmokers' rights, majority will, and the need to protect worker health and the public health.

## Results

We begin by summarizing the nature of legislation in each state (see Table 1 for the specifics of each law), then review the nature of arguments made in the six states. We conclude by testing the strength of Schattschneider's theoretical claims.

**Table 1:** Details of state legislation, 1992–2003

*State*	*Year*	*Process*	*Policy details*
Utah	1994	Task force and legislative hearings	*Private worksites: *Smokers and nonsmokers in separate areas *Restaurants: *Smoking banned *Bars: *No restrictions
South Dakota	2002	Legislative hearings	*Private worksites: *Smoking banned *Restaurants: *Smoking allowed where alcohol is served *Bars: *No restrictions
Florida	1992	Legislative hearings	*Private worksites: *Smokers and nonsmokers in separate areas *Restaurants: *Requires separate areas if restaurant seats more than 50 people *Bars: *No restrictions *(Also preempts stronger local laws)*
Oregon	2002	Legislative hearings	*Private worksites: *Smokers and nonsmokers in separate areas *Restaurants: *Requires separate areas if restaurant seats more than 30 people *Bars: *No restrictions *(Also preempts stronger local laws)*
North Dakota	2003*	Legislative hearings	*Private worksites: *Would have banned smoking *Restaurants: *Would have banned smoking *Bars: *Proposed no restrictions
Louisiana	1993	Legislative hearings	*Private worksites: *If nonsmokers object to smoking, employers should try to accommodate both smokers and nonsmokers *Restaurants and bars: *No restrictions *(Also preempts stronger local laws)*

Source: Proposed and final legislation.

* North Dakota proposed but did not pass new workplace smoking restrictions.

Note: States ordered from strongest legislation to weakest legislation; North Dakota is ranked based on its existing law

### History of state legislation and outside participation

Each state that considered a statewide workplace smoking law had a slightly different process, although all the states held hearings and engaged in legislative debate. Some state hearings drew testimony from national advocacy groups or tobacco industry lobbyists. Participants discussed issues such as the importance of protecting public health, whether a law was necessary or whether businesses would self-regulate, the rights of smokers and nonsmokers, the possible economic impact of a new law, and the relevance of public opinion. The types of participation and argument in each state are summarized in Table 2.

**Table 2:** Summary of participation and issues discussed, by state (ordered from strongest to weakest legislation)

**State**	**Year initiated**	**Outside participants**	**Participant types**	**Major issues discussed**
Utah	1993	65	Local and national advocates, including former Surgeon General C. Everett Koop	Protecting public health; risks of secondhand smoke; economic impact of a new law; state reputation for excessive moralizing
South Dakota	2001	29	Local and national advocates, including lobbyists for Brown & Williamson and RJ Reynolds	Protecting public health; whether legislation was needed or businesses would self-regulate; personal experiences with smoking
Florida	1992	42	Local advocates and some national organizations	Protecting public health; whether legislation was needed or businesses would self-regulate; smoker and nonsmoker rights
Oregon	2001	28	Local advocates and some national organizations	Pre-emption clause (whether the state should prevent localities from passing more-restrictive laws); protecting public health; the views of state residents; potential negative economic impacts of a law
North Dakota	2001	12	Local advocates and constituents	Whether government action was necessary or businesses would self-regulate; personal experiences with smoking; limited discussion about protecting public health
Louisiana	1992	3	Tobacco industry lobbyist and legislative staffers	Importance of accommodating smokers; difficulty of enforcing smoking restrictions

Source: Transcribed testimony given for and against proposed and final legislation, coded by the authors.

To a certain extent, these major points of discussion suggest some of the broad cultural differences between these states. The state of Utah is largely Mormon, and more willing to legislate on social issues than many other states in the western US. Like those states, however, the Utah state government has typically been cautious about imposing economic regulations. North Dakota and South Dakota, which have similar state cultures, both proposed very strong clean indoor air laws around the same time, but only South Dakota's passed. Overall, if smoking legislation were associated with historical willingness to legislate on economic and public health issues, we would have expected Utah and Oregon to pass the strongest legislation, and the Dakotas to pass (or fail to pass) the weakest legislation, but with the exception of Utah, this was not the case. Unfortunately it is difficult to judge how these cultural differences might motivate greater or lower degrees of testimony; for example, many states in the western US have legislatures that meet only every two years, and the limited time that legislators are in session could either motivate or discourage participation.

### Types of ideological arguments

We classified the ideological arguments in each state as privatizing or socializing. Examples of each type of argument are provided in Table 3. Privatizing arguments focused on whether government involvement was appropriate or on the need to maintain status quo protections of smokers. Free enterprise arguments emphasized the right of individuals and businesses to make their own decisions. In contrast, arguments about smoker's rights made the claim that establishing non-smoking workplaces, or even non-smoking areas in public places, would discriminate against smokers. Similarly, arguments about courtesy to smokers claimed that further legislation would unfairly impose on smokers. Overall, these kinds of ideological arguments took the question of workplace smoking restrictions as one related to the rights of individuals and the appropriate scope of government action.

**Table 3:** Examples of ideological arguments by type

**Argument type**	**Quote**
***Privatizing***: Free enterprise	"Leave the free enterprise system to go on its own. The market'll take care of it. We don't have to legislate. This body doesn't have one cent invested in any of these businesses. The state doesn't have a penny invested in the businesses. Who are we to tell these people how to run their businesses? But we're only telling a certain few how to run their businesses. Let the people run their own businesses."
	"Our customers are adults, over twenty-one years old. Legislation should not decide this issue, the market should. Laissez-faire capitalism is the foundation of our market system. People will spend their money at the businesses that treat them right and cater to their needs."
***Privatizing***: Smoker's rights	"If we're making restaurants, and presently you're all smoking, have a no-smoking section, we ought to make those that are presently non-smoking have at least a small section for smoking. It's fair and equal and fair. And we shouldn't be discriminating against smokers where we are in this bill."
***Privatizing***: Courtesy (fairness to smokers)	"But, you have to say one thing about smokers as a group, they are paying more than their fair share of taxes. And by god when you're paying more than your fair share and we're already limiting them to where they can sit and everything else in restaurants. I think they have been punished enough."
***Socializing***: Equal protection (for nonsmokers)	"If you want to smoke it just seems to me as if you should have the right to do so. But if I choose not to smoke I should have the right to not have to. I should have the right to not have to associate with Senator Landry if I don't want to, is the point that I'm making."
***Socializing***: Public opinion	"Now if I was going to move ahead a little bit farther I might try to make an argument about that maybe we should take into consideration about what Oregonians actually think about this issue. Well, from a relatively recent poll, 82% of Oregonians say that people should be protected from second hand smoke."
***Socializing***: Protect public health	"It would be our view that business has a responsibility to provide a healthy and safe work environment for employees and customers and that the right to do business in this state comes with responsibilities, responsibilities that this body imposes on business every day after weighing the costs and benefits to the citizens of this state. And I mean all the citizens of this state, not just those lucky enough to be business owners in the State of South Dakota."

Source: Transcribed testimony given for and against proposed and final legislation, coded by the authors.

In contrast, ideological arguments that were socializing in nature either directly challenged the claim of individual rights by asserting the rights of nonsmokers or called upon government to respond to majority will and to protect the health of the public. The most common argument was that state government has the responsibility to protect the health of workers and the public. Overall, socializing ideological arguments looked at new workplace smoking restrictions as a way to protect public health, prove government responsiveness, and establish equal rights for nonsmokers.

### Analysis of arguments

Our analysis compares the number of participants, the share of ideological arguments as a percentage of all the types of arguments coded (relative to scientific, economic, and political claims), and the nature of ideological arguments to the eventual strength of legislation passed in each state.

### Hypothesis 1: Stronger legislation associated with more participation

**Figure 1:**
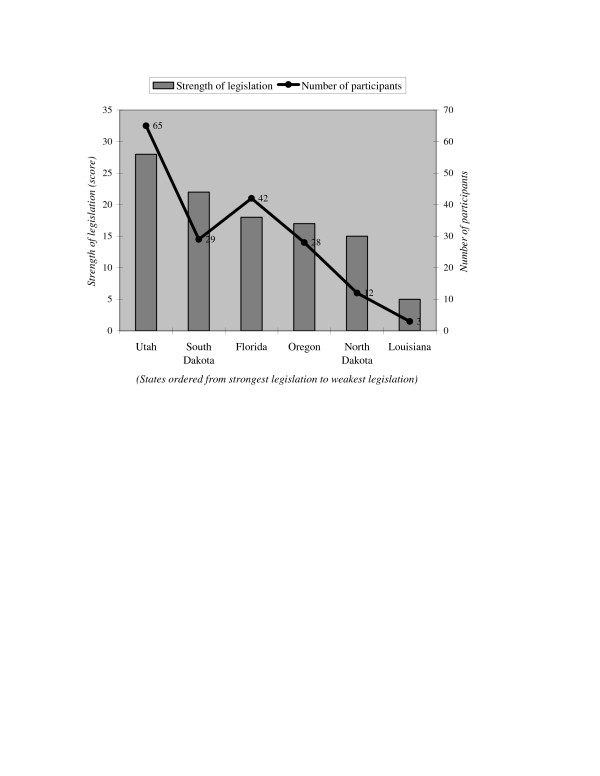
**Outside participants in workplace smoking legislative hearings, 1992–2003**. Source: Coding of committee hearings, floor debates, task force meetings, and letters introduced as testimony. Details of the coding described in the text.

Schattschneider argued that the greater the participation by outsiders, the more likely it should be that an outcome reflects public will. Increased participation should be unpopular with individuals and groups that benefit from the status quo. Our review of workplace smoking legislation confirms this supposition. With one exception, our descriptive data suggest that the more outsiders who participated in a state's legislative process, the stronger the eventual legislation (see Figure 1).

Outside participants in the states we considered represented a range of occupations, including business owners, restaurant and bar workers, physicians, public health advocates, tobacco industry lobbyists, consultants, and legislative aides. Utah passed the strongest Clean Indoor Air Act, and the number of individual non-legislators (65) who participated in the process exceeded by a third that of Florida, the state with the second highest number of participants. In contrast, Louisiana, which passed the weakest legislation, had only three outside participants involved in the debate over a new clean indoor air law. Outside participation in most of the other states fell between these two extremes. Only Florida appears exceptional, with many more non-legislators (42) participating than there were in South Dakota (29), which eventually passed a much stronger law. We believe that this discrepancy reflects the fact that most states considered their workplace smoking legislation over a two to three year period, but Florida's debate spanned almost six years. Overall, the association between the number of outside participants and the strength of legislation is consistent with our expectations. Although limited sample size makes it impossible to test all alternative hypotheses, participation in the legislative process appears to be related to legislative outcomes. Notably, despite  decreased public willingness to tolerate secondhand smoke exposure over  the course of this decade, both the strongest and weakest laws were  introduced at the beginning and the end of the time period we  considered, suggesting that this change in public opinion may have  little weight unless it is expressed directly to legislators.

### Hypothesis 2: Stronger legislation associated with less ideological focus

**Figure 2:**
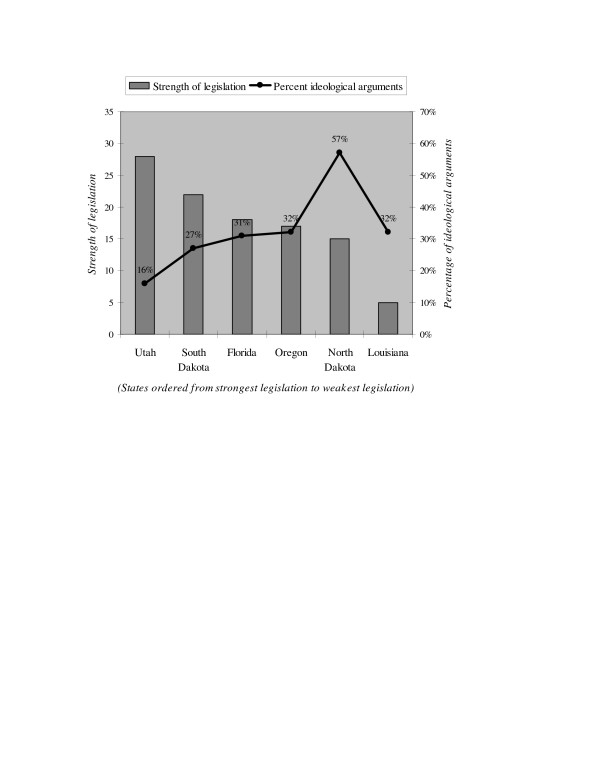
**Strength of legislation and percentage of arguments relating to ideology in workplace smoking legislation testimony, 1992–2003**. Source: Coding of committee hearings, floor debates, task force meetings, and letters introduced as testimony. Details of the coding described in the text. Note: Percentage of ideological arguments represents the number of passages coded as ideological over the total number of all passages coded.

We also considered the question of whether stronger workplace smoking legislation was associated with less emphasis on ideological arguments, relative to other coded arguments. The relationship is less obvious, though still apparent (see Figure 2). As expected, the percentage of ideological arguments, as a percentage of all arguments, was lowest in Utah (16%), which had the strongest legislation. However it was highest in North Dakota (57%), which had only the second weakest legislation (as noted, this ranking refers to the existing law rather than the proposed legislation). Moreover, the use of ideological arguments is similar in South Dakota (27%), Florida (31%), and Oregon (32%), even though the strength of their legislation differs.

Louisiana is an obvious exception, reflecting the behavior of one of the few participants in Louisiana's legislative hearings: a tobacco industry lobbyist. She rewrote the bill nearly single-handedly, and spent substantial time talking about details such as enforcement measures (which were nearly eliminated). She used ideological arguments only when legislators questioned her about the justification for changes (all of which favored the tobacco industry), and her usual response was that it was "fair" to guarantee that people could smoke in the workplace.

Overall, our evidence suggests that less focus on ideological arguments is associated with stronger legislation, though further research is needed. In our coding, a smaller share of ideological arguments meant that there was more focus on scientific, economic, or governmental arguments. Except in Louisiana, using ideological arguments appeared to be associated with weaker legislation. A greater focus on non-ideological arguments was evident in states that passed stronger legislation.

### Hypothesis 3: Stronger legislation associated with more socializing arguments

**Figure 3:**
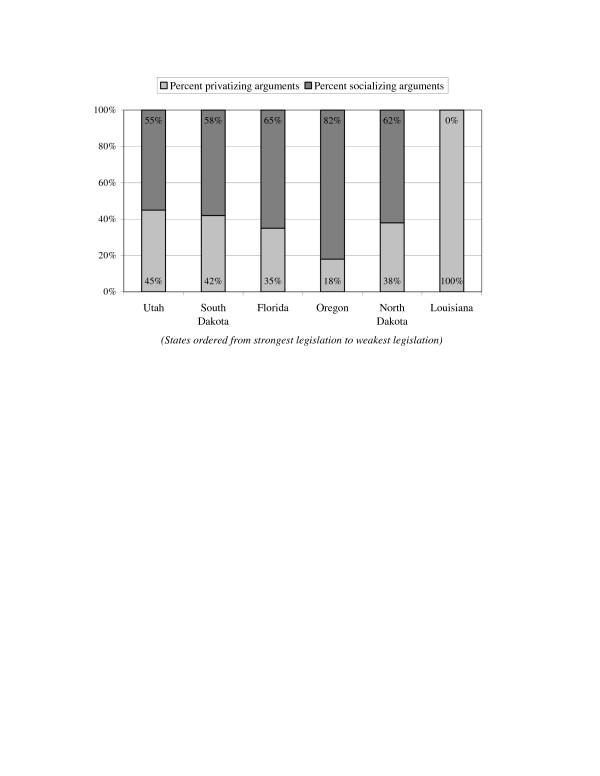
**Privatizing versus socializing ideological arguments in workplace smoking legislation testimony, 1992–2003**. Source: Coding of committee hearings, floor debates, task force meetings, and letters introduced as testimony. Details of the coding described in the text. Note: Percentage of privatizing arguments represents the number of passages coded as privatizing over the total number of all ideological passages coded; percentage of socializing arguments is the reverse.

Finally, we considered whether stronger workplace smoking legislation was associated with the greater use of socializing arguments and lesser use of privatizing arguments. We found that Schattschneider's case was weakest here, though our results may be confounded somewhat by differences in state political culture or history that make legislators more responsive to different types of arguments[58] Although Louisiana, the state with the weakest legislation, had by far the largest share of privatizing arguments (100%), both Utah and South Dakota participants used more privatizing arguments than participants in North Dakota's legislative process. With the exception of Louisiana, none of the states followed the hypothesized pattern (see Figure 3). Our evidence does not support Schattschneider's contention that the use of socializing arguments is associated with outcomes that are more oriented to collective goals.

## Conclusion

Our research suggests two major conclusions. First, our analysis of legislation on workplace smoking restrictions validates Schattschneider's long-standing assertion about the relationship between participation and political outcomes. Increased participation in the legislative process does appear to be related to legislative outcomes supportive of public health. Second, these findings suggest some practical strategies that may be effective in future efforts to pass public health legislation. Public health advocates should make efforts to appear at legislative hearings and give testimony, or to encourage legislators to hear a wide range of views when considering new legislation by establishing task forces that explicitly solicit public opinion. Existing research notes that health professionals are widely viewed as persuasive by legislators, and that policy makers would like more contact with them [59-61] Moreover, recent research on advocacy and lobbying before legislatures suggests that tactics used in the United States may also be applicable to efforts in other democracies[62,63]

Although Schattschneider's claims about bias in political representation have been widely accepted for decades, very little research attempts to test whether greater participation is correlated with different political outcomes. In part, this reflects the focus of political scientists on national-level policy making and the emphasis of public health researchers on case studies of individual countries, laws, or states. In either type of study it is difficult to observe multiple iterations of the same decision under different circumstances at the same time. In contrast, our review of state Clean Indoor Air Acts offered the opportunity to consider legislation on a single issue in different forums over the course of approximately a decade. These data, however, suffer certain limitations. Because not all states keep records of their legislative activities, it was only possible to review the activities of a subset of legislatures, which prevents us from controlling for factors such as differences in population or political culture. In addition, because states do not always consider legislation on the same issues contemporaneously, our analysis is limited to the passage of one kind of legislation, clean indoor air laws. Finally, over the course of the decade we considered public opinion about risks of exposure to secondhand smoke changed substantially, although our findings do not show increased policy strength over time. Nonetheless, the analysis is illustrative.

Future research that could review testimony over a larger number of localities, states, or countries and a more limited time period would allow for additional controls that would strengthen this finding. Although exogenous events that trigger multiple policy changes across jurisdictions are rare, they offer a unique opportunity for researchers and advocates who wish to understand what motivates policy change. In addition, additional research on participation patterns in different states across a range of issues and over time could shed light on the question of whether certain state cultures or the presence of local advocacy groups inspire citizens to testify more frequently.

Schattschneider claimed that full participation would produce outcomes that served the public interest, and our research provides support for this claim. However, it is also possible that legislators who are already willing to accept stronger public health legislation were also more willing to hear more testimony, because they correctly assessed that public opinion supported strong legislation. In either case, our results offer guidance to public health advocates. If greater participation leads to stronger legislation, then attendance at legislative hearings may increase the likelihood of passing legislation that benefits public health. If legislators who are willing to pass stronger legislation are willing to hear a range of public comment, then observing the rules and practices that legislators establish when considering new legislation, specifically the degree to which testimony is allowed and encouraged, can predict the likelihood of success. Even in the absence of relevant legislation, our findings suggest that public health advocates should also support proposals to encourage open public hearings.

In keeping with evidence suggesting the importance of issue framing,[18,64] the passage of stronger legislation appeared to be associated with less focus on ideology, and more focus on the substance of legislation. We conclude that public health advocates may also be more successful if they focus on public health concerns rather than being drawn into ideological discussion, despite the suggestion of some researchers than tobacco control advocacy focus on claims of individual rights[65]

Although seeking to pass public health legislation (rather than regulate or litigate) is particularly relevant in the case of tobacco, where legislative changes are most effective in modifying behavior,[66] the lessons drawn from tobacco control policy changes can be applied to other public health problems, such as obesity and alcohol consumption[67] The specific actions our research suggests are effective, such as attending public hearings, are relatively low cost in comparison to other political activities such as hiring lobbyists and making campaign contributions, and these actions appear to be ultimately more effective in establishing public health legislation[38,68]

## Competing interests

The author(s) declare that they have no competing interests.

## Authors' contributions

D.E. Apollonio conceived the paper topic, assisted with the study and coding, conducted the analysis, and drafted the majority of the manuscript. P. Lopipero assisted with the study and coding and drafted the methods section. L.A. Bero supervised the study and edited the manuscript. All authors read and approved the final manuscript.
